# Peri-Intubation Cardiac Arrest in the Pediatric Emergency Department: A Novel System of Care

**DOI:** 10.1097/pq9.0000000000000365

**Published:** 2020-10-26

**Authors:** Erin F. Hoehn, Preston Dean, Andrew J. Lautz, Mary Frey, Mary K. Cabrera-Thurman, Gary L. Geis, Erika Stalets, Matthew Zackoff, Tena Pham, Andrea Maxwell, Adam Vukovic, Benjamin T. Kerrey

**Affiliations:** From the * Division of Emergency Medicine, Cincinnati Children’s Hospital Medical Center, Cincinnati, Ohio; † Children’s Hospital of Pittsburgh of UPMC, Division of Emergency Medicine, Pittsburgh, Pa.; ‡ Department of Pediatrics, University of Cincinnati College of Medicine, Cincinnati, Ohio; § Division of Critical Care Medicine, Cincinnati Children’s Hospital Medical Center, Cincinnati, Ohio; ¶ Emergency Services, Cincinnati Children’s Hospital Medical Center, Cincinnati, Ohio

## Abstract

Patients with physiologic disorders, such as hypoxemia or hypotension, are at high risk of peri-intubation cardiac arrest. Standardization improves emergency tracheal intubation safety, but no published reports describe initiatives to reduce the risk of cardiac arrest. This initiative aims to improve the care of children at risk of peri-intubation cardiac arrest in a pediatric emergency department (PED). We specifically aimed to increase the number of patients between those with peri-intubation cardiac arrest by 50%, from a baseline of 11–16, over 12-months.

**Methods:**

Our multidisciplinary team outlined a theory of improvement and designed interventions aimed at key drivers. The primary intervention was creating a PICU-ED Team (PET) and a checklist to guide the assessment and mitigation of risk for peri-intubation arrest and rapid consultation of the pediatric intensivists. The PET was iteratively refined, and we collected data by a video review of tracheal intubations.

**Results:**

Fifty-one patients with risk factors for peri-intubation arrest underwent tracheal -intubation in the PED from January 2016 to March 2020: 14 with PET activation since PET go-live in April 2019. None of the 14 PET patients had a peri-intubation cardiac arrest. Ninety-three percent (13/14) of PET patients were intubated in the PED, and 78% (10/13) of these patients had the first intubation attempt completed by PED physicians (balancing measures).

**Conclusion:**

We successfully developed the PET to mitigate the risk of peri-intubation cardiac arrest without significantly reducing key procedural opportunities for the PED. Initial data are promising, but further refinement is needed.

## INTRODUCTION

Peri-intubation cardiac arrest is the most feared complication of emergency tracheal intubation, but the incidence in children is incompletely described. Peri-intubation cardiac arrest accompanies approximately 1.7% of intubations in the pediatric intensive care unit (PICU) setting,^1^ but the national rate in the pediatric emergency department (PED) is unknown. Anatomically difficult airways are known to be associated with adverse events.^1^ Patients can also have physiologically difficult airways, with physiologic derangements sufficiently severe to increase the risk of cardiovascular collapse during airway management.^2^ Emerging evidence suggests the physiologically difficult airway is also associated with increased risk of adverse events including cardiac arrest.^1,3^

Cardiac disease, hypoxemia, and hypotension have previously been identified as risk factors for peri-intubation cardiac arrest.^1,4,5^ Identifying these risk factors is necessary but insufficient to mitigate the risk of peri-intubation cardiac arrest. Since physiologically high-risk pediatric intubations are low-frequency events,^6^ a system-level improvement process is needed to decrease the risk of mortality. Some investigators have shown that quality improvement initiatives can reduce peri-intubation adverse events, specifically hypoxemia.^7–9^ The use of QI methodology in the PED to mitigate the risk of peri-intubation cardiac arrest has not been reported.

In 2018, an unfortunate case of peri-intubation cardiac arrest in our institution’s PED prompted further investigation. Before this case, previous studies at our institution identified an intubation-associated cardiac arrest rate of 1.8% (2/114) over 12 months.^10^ While this rate is similar to published data for the PICU, due to issues identified in the case review and the high mortality associated with peri-intubation arrest, we felt it to be unacceptable. We embarked on improvement initiatives to eliminate this phenomenon.

The long-term objective of this improvement initiative is to minimize the risk of peri-intubation cardiac arrest in our PED. We theorized that the infrequent nature of these patients was a critical barrier to pre-intubation recognition and mitigation. We also theorized that a standardized approach would facilitate the early identification of high-risk patients and mitigate the risk of arrest. We intend to describe the PICU-ED Team (PET) development, a novel response system designed to improve the care of patients at high-risk for peri-intubation cardiac arrest. Our primary aim is to decrease the frequency of peri-intubation cardiac arrests, as measured by a 50% increase in the number of high-risk patients between patients with peri-intubation arrest, over 12 months. Our secondary aim is to assess the impact of the intervention on post-intubation cardiac arrest at any time in PED care and monitor the discipline of the proceduralist and location of tracheal intubation (PED versus PICU) as balancing measures.

## METHODS

### Setting

We conducted this project in the resuscitation area [shock trauma suite (STS)] of a high-volume academic PED with an annual volume of 62,000 visits per year. The STS has approximately 4,500 patient encounters per year or 7% of the total patient volume. The parent institution is a level I trauma center, with approximately 85% of the regional pediatric admissions from a population base of more than 2,000,000 people.

The institution trains approximately 4 pediatric emergency medicine (PEM) and 4 critical care (CC) fellows per year. Our institutional review board determined the project to be non-human subjects research and, therefore, did not require institutional review board review and approval.

Approximately 100 patients per year are intubated in the PED. All tracheal intubations occur in the STS. Each of the 4 STS bays is equipped with ceiling-mounted digital video cameras and microphones that record continuously. Video recordings of patient encounters are available for review using a proprietary software program (Live Capture, B-Line Medical, Washington, DC). Patients and families provide consent for video recording in the general PED consent to treat form.

Since 2013, the PED has had a well-established process of both quality assurance through structured video review and a clinical pathway, including the use of rapid sequence intubation (RSI) checklist. Video-based studies have found the checklist, and other vital processes are performed for more than 90% of patients undergoing RSI.^9^ Multiple prior projects have demonstrated sustained improvement in the safety of tracheal intubation in our PED.^9,11^

### Interventions

#### Improvement Team

In August 2018, PEM and CC faculty physicians initiated a project to reduce the risk of peri-intubation cardiac arrest among high-risk patients in our PED. The improvement team consisted of 6 faculty (4 PEM and 2 CC), 2 fellows (1 PEM and 1 CC), and 3 nurses (2 ED and 1 CC).

#### Theory and Key Drivers

**Fig. 1.**
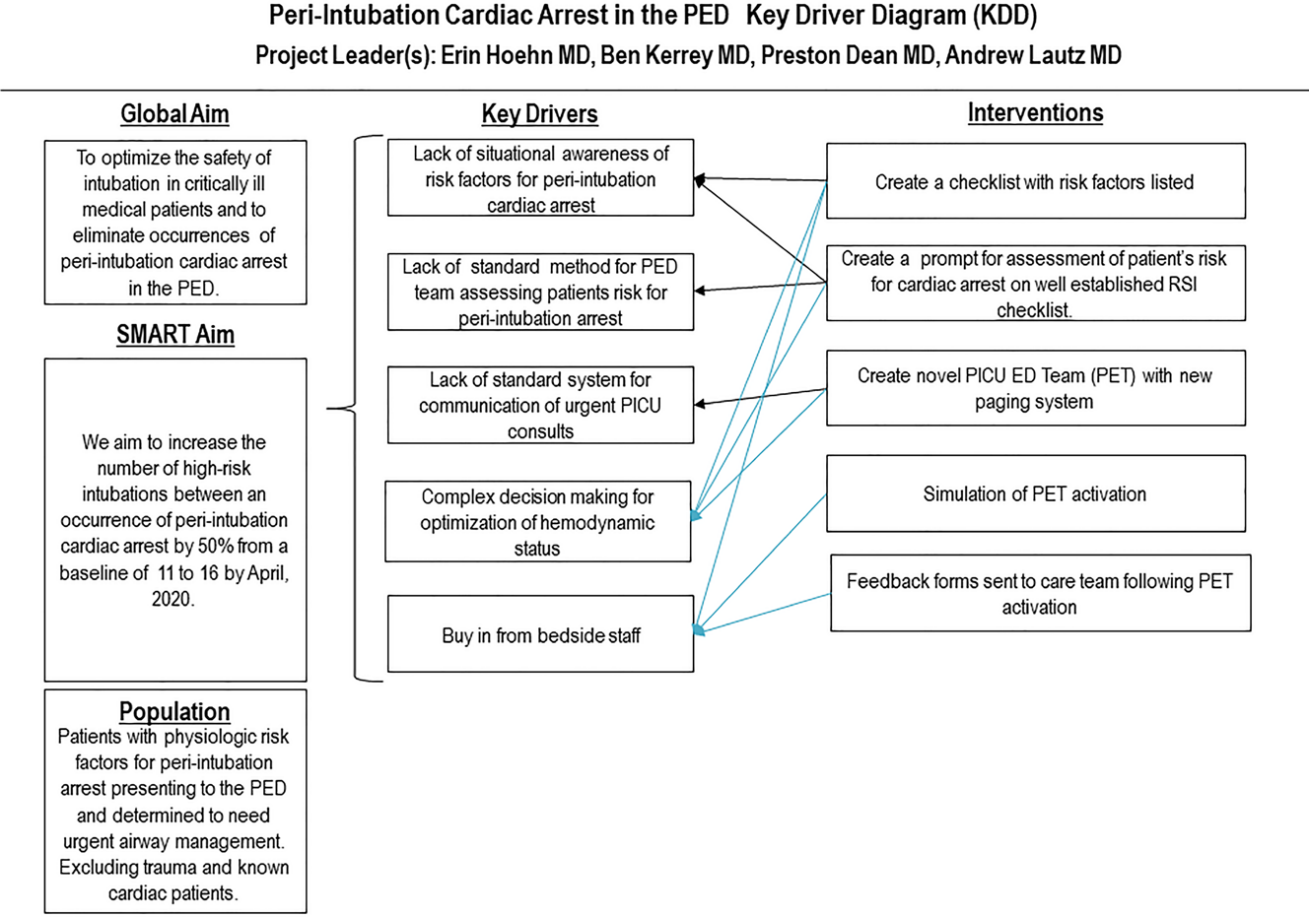
Key driver diagram. KDD, key driver diagram.

The improvement team first developed a theory of improvement based on extensive knowledge of our airway management system and additional video review of actual cases, including the index patient. We theorized the risk of peri-intubation cardiac arrest could be minimized by interventions that addressed the following key drivers: (1) improved situational awareness of higher-risk patients by the PED team; (2) thoughtful optimization of patient hemodynamic status before tracheal intubation; and (3) facilitation of efficient PICU arrival to the PED and effective communication between PED and PICU clinicians. The improvement team constructed a key driver diagram to make explicit the theory for improvement (Fig. 1).

#### PICU-ED Team

The primary intervention was the development of the novel PET. The PET consisted of the primary PED team for the patient and a senior PICU fellow or attending and PICU nurse who responded to the PED. The PED team was responsible for identifying children at high-risk for peri-intubation arrest, using specific PET criteria, and activating the PET through a team-specific page.

#### PET Criteria

The improvement team determined situational awareness of risk factors was a key driver of peri-intubation cardiac arrest. To address this driver, the improvement team developed high-risk criteria that would trigger PET activation. The improvement team drafted an initial list of criteria based on existing literature;^1,4,5^ reviews of the ten most recent cardiac arrest cases within 4 hours of PICU admission, and more than 10 years of video-based case reviews of tracheal intubations in the PED. The improvement team then revised the criteria by applying them to a historical cohort of 20 STS patients who had undergone tracheal intubation in the PED and group discussion, which continued until consensus was achieved. The final list of high-risk PET criteria was: (1) hypotension for age, (2) clinical concern for cardiac dysfunction, (3) persistent hypoxemia (pulse oximetry < 90%) despite supplemental oxygenation or positive pressure, (4) severe metabolic acidosis (pH < 7.1), (5) post-return of spontaneous circulation, and (6) status asthmaticus.

#### Process Checklist and Simulation

**Fig. 2.**
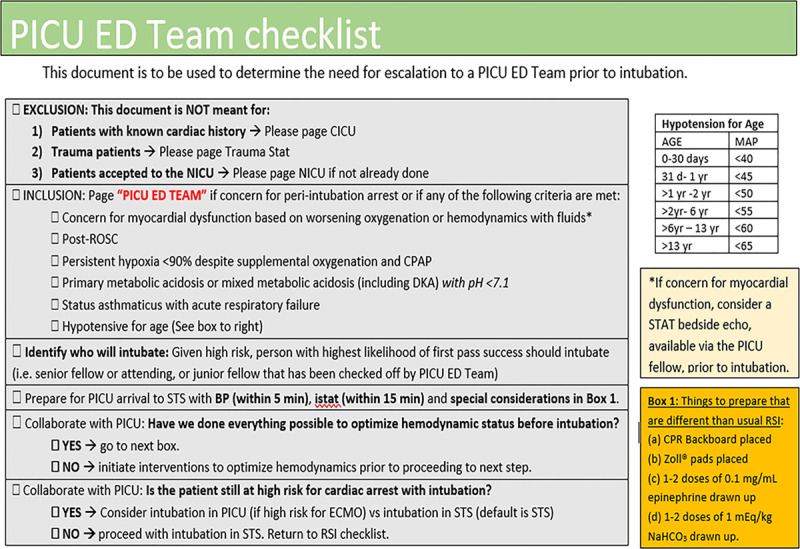
Novel PICU-ED Team checklist. BP, blood pressure; CICU, cardiac intensive care unit; CPAP, continuous positive airway pressure; DKA, diabetic ketoacidosis; I-stat, venous blood gas; MAP, mean arterial pressure; NICU, neonatal intensive care unit; ROSC, return of spontaneous circulation.

The improvement team designed a process checklist (Fig. 2) that included (1) the high-risk criteria, (2) information to promote effective communication, (3) prompts to discuss optimization of hemodynamics, and (4) suggested cardiac arrest precaution measures. The PET checklist was placed on the reverse side of the existing RSI checklist.^9^ A prompt was added to the RSI checklist to assess for high-risk status.

To promote effective communication with PICU, the CC members of our improvement team identified the critical aspects of a patient’s care they need to understand quickly upon arrival to the PED. Based on this input, we added the checklist prompts for communication to the PICU providers regarding the most recent blood pressure and venous blood gas results. To mitigate risk factors for cardiac arrest, we added checklist prompts that optimize hemodynamics and suggest cardiac arrest precaution measures (eg, defibrillator pads on the patient, backboard down). The improvement team believed high-risk patients have complex and varied pathology leading to their physiologic derangements. Patients may need different therapies to optimize hemodynamics before intubation, depending on the underlying pathology. Therefore, the checklist does not contain specific hemodynamic interventions. Instead, the checklist includes prompts to promote a thoughtful patient-specific discussion before intubation.

The PET elements were iteratively tested and refined through a series of ten in situ simulations conducted over 6 months. In concert with our institution’s Center for Simulation and Research, simulation scenarios were designed to test the identification of high-risk patients and PET processes. Improvement team members facilitated each simulation, and recorded observations were discussed by the larger team to refine the process.

#### Education and Dissemination

We focused educational efforts on critical groups including, PEM and CC fellows, PED respiratory therapists, and PED and CC nurses through regular divisional meetings and emails. Debriefings following in situ simulations increased awareness of and reinforced the goals of the PET.

### Study of the Intervention

PET-eligible patients were children presenting with a critical illness needing emergency tracheal intubation. We excluded critically injured (trauma) patients since the institutional trauma system includes PICU, trauma surgery, and anesthesiology team members. Additionally, we excluded children with known cardiac disease, as they are typically co-managed with cardiac intensivists. Finally, we excluded crash or no-medication intubations, as these patients are typically already in cardiac arrest.

We identified eligible patients and PET activations through our existing STS quality assurance program, including a database of all STS activations. The STS database is based on daily reports generated from our institution’s electronic health record, capturing patients undergoing tracheal intubation in the PED with high reliability.^10^ The lead improvement team nurse (M.F.) screened the database for eligible patients and PET activations. We reviewed all eligible RSI cases to determine if any PET criteria were present. If a likely missed eligible case was identified, an improvement team physician (P.D.) reviewed the corresponding patient video. Two members of the improvement team (P.D. and G.L.G.) performed structured video-based data collection for each PET activation. The improvement team then met monthly to review activation data and potentially missed cases, discussed issues with each activation, and made necessary revisions to the PET criteria/system. After each activation, the team emailed a feedback form to the care team members, requesting additional feedback on the PET process.

To establish a baseline for the frequency of peri-intubation arrest among patients meeting high-risk criteria, we queried the STS database for all patients meeting above inclusion and exclusion criteria for the 39 months from January 1, 2016 to March 31, 2019. We conducted an additional structured review of the electronic health record to collect data not available in the STS quality assurance database. We identified a subset of patients considered high-risk intubations if they met one or more previously defined high-risk PET criteria.

### Measures

Our global aim is to improve the care provided to critically ill children needing emergency airway management by reducing the risk of peri-intubation cardiac arrest. The primary outcome measure, though rare, is a peri-intubation cardiac arrest in a patient meeting one or more high-risk criteria. Peri-intubation cardiac arrest is defined as cardiac arrest (documented/observed chest compressions or asystole/pulseless electrical activity) within 10 minutes of tracheal intubation. This timeframe is consistent with definitions in the literature, which vary from 5 to 20 minutes.^1,4,12–14^ The secondary outcome measure is a post-intubation cardiac arrest in the PED. Post-intubation cardiac arrest is defined as a cardiac arrest that occurred while the patient was still in the PED, regardless of time elapsed after intubation. This term includes all peri-intubation arrests (within 10 minutes of intubation) plus patients with cardiac arrest outside of that timeframe but while still in the PED.

As process measures, we tracked both instances when a patient met PET criteria in which the team was not activated and those in which a team activation was initiated without any PET criteria being met. Balancing measures were (1) the discipline of the proceduralist and (2) the location of tracheal intubation (PED versus PICU). These balancing measures were chosen due to the concern that the PET’s implementation may have the undesired consequence of limiting opportunities for intubation by PED physicians.

## ANALYSIS

We tabulated all activation data and generated standard descriptive statistics. All PET activations, any missed eligible activations, and key clinical features and outcomes of these cases were described. Annotated control charts were developed and updated regularly to monitor outcome measures. We used G charts to track these measures as high-risk intubations are in themselves rare; even more rare is the occurrence of either peri- or post-intubation cardiac arrest. G charts track the number of successful events between rare-event failures. When using G charts, data are skewed, and there is no lower control limit; thus, a theoretical median is used for the centerline, which is calculated by the equation 0.693 × mean.^15^ We used standard rules for interpreting a Shewhart chart to determine special cause variation indicating the association between PET development and significant changes in the process or outcomes.^15^

## RESULTS

Fifty-one patients with risk factors for peri-intubation arrest underwent tracheal intubation in the PED from January 2016 to March 2020: 36 in the historical cohort and 15 since PET go-live in April 2019. Ninety-three percent (14/15) of high-risk patients intubated after go-live had PET activation (Table 1). One patient met criteria yet had no PET activation (missed eligible), and 1 had PET activation yet did not meet criteria (false positive). From PET go-live in April 2019 to March 2020, 81 PED non-trauma patients have required tracheal intubation in the STS. None of the 81 patients had peri- or post-intubation cardiac arrest.

**Table 1.** PET Activations (April 2019 to March 2020)

Age	Diagnosis	PET Criteria Met	Loc	Checklist Used	Interventions Before RSI	Arrest Pre-cautions	Intubation Success	CPR Post-RSI	Survival
9 y	Septic shock	Hypoxia	ED	No	IVF	Defib pads CPR board	Yes	No	Yes
3 m	Status Epilepticus	Metabolic Acidosis	ED	Yes	IVF	None	Yes	No	Yes
11 m	Duodenal Web	Hypotension	ED	Yes	IVF	None	Yes	No	Yes
8 m	Status Epilepticus, HIE	Metabolic Acidosis	ED	Yes	IVF	None	Yes	No	Yes
7 d	Septic shock	Metabolic Acidosis, Cardiac Dysfunction, Hypotension	ED	Yes	IVF Vasopressor	None	Yes	No	No
22 m	Status Epilepticus	Resp acidosis w/ difficulty ventilating*	ED	Yes	IVF	CPR board	Yes	No	Yes
10 d	Septic shock	Metabolic Acidosis, Hypotension	ED	Yes	IVF Vasopressor	Defib pads CPR board	Yes	No	No
36 y	Respiratory Arrest	Post-ROSC, Hypoxia, Cardiac Dysfunction concern	ED	No		Defib pads CPR board	Yes	No	Yes
16 y	Respiratory Arrest	Hypoxia, Hypotension	ED	No	IVF	None	Yes	No	Yes
10 y	Respiratory Failure	Hypoxia, Hypotension	ICU	Yes	IVF Vasopressor	Defib pads	Yes	No	Yes
9 y	AMS	Hypotension	ED	Yes	IVF Vasopressor	Defib pads CPR board	Yes	No	Yes
2 y	Pneumonia	Hypoxia	ED	Yes	IVF		Yes	No	Yes
3 m	Drowning	Post-ROSC, Cardiac Dysfunction	ED	Yes	IVF Vasopressor	Defib pads CPR board	Yes	No	No
3 m	Pneumonia	Cardiac Dysfunction, Metabolic Acidosis	ED	Yes	IVF	Defib pads	Yes	No	Yes

List of demographic data, clinical variables, interventions, and outcome data for all PET team activations from April 2019 to March 2020.

*Did not meet formal PET Criteria, but activated due to provider concern.

AMS, altered mental status; CPR, cardiopulmonary resuscitation; Defib pads, defibrillator pads; ED, emergency department; HIE, hypoxic-ischemic encephalopathy; IVF, intravenous fluid resuscitation; Loc, location of Intubation; ROSC, return of spontaneous circulation.

**Fig. 3.**
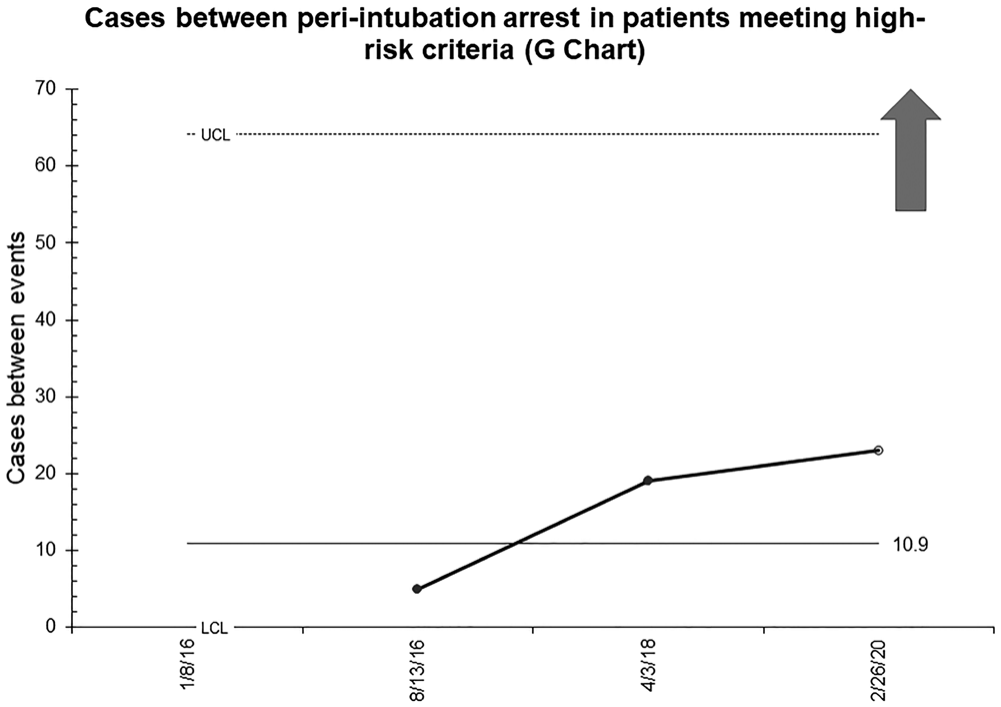
Peri-intubation arrest G-chart: Patient encounters of high-risk intubations between events of cardiac arrest within 10 minutes of intubation.

Figure 3 demonstrates the primary outcome measure of the number of patients with at least 1 high-risk criteria intubated between episodes of the peri-intubation arrest. Although preliminary, the theoretical median is currently approximately 11 high-risk intubations between cases of peri-intubation arrest. A trial upper control limit has been set, given the rare nature of this outcome and the limited number of data points. There have been 24 cases since the last high-risk intubation resulting in peri-intubation arrest (dating to before PET go-live); the last data point on the graph is an open circle to signify that the patient on February 26, 2020 did not experience a peri-intubation arrest and that this count is ongoing.

**Fig. 4.**
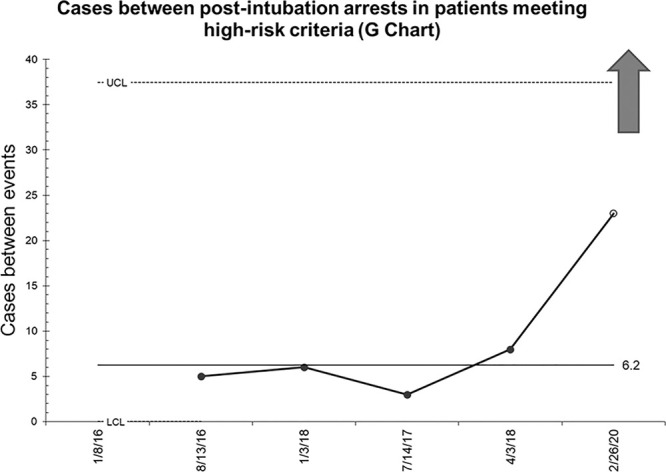
Post-intubation arrest G-chart: encounters of high-risk intubations between events of cardiac arrest following intubation at any time point while the patient is still in the PED.

Figure 4 demonstrates the secondary outcome measure of the number of patients with at least 1 high-risk criteria intubated between episodes of post-intubation arrests. The theoretical mean is currently approximately 6 high-risk intubations between post-intubation arrests. A trial upper control limit is again set. There were no post-intubation arrests that occurred more recently than the most recent peri-intubation arrest. Therefore, there have also been 24 cases since the last high-risk intubation resulting in a post-intubation arrest.

For balancing measures, 93% (13/14) of PET-activation patients were successfully intubated in the PED. Seventy-eight percent (10/13) of patients intubated in the PED had a first intubation attempt by a PED physician (resident, fellow, or attending). Otorhinolaryngology or anesthesiology physicians intubated the remaining 3 patients. The PICU intubation resulted from a transfer to the PICU for tracheal intubation to have extracorporeal membrane oxygenation therapy available if needed; PICU fellow successfully intubated this patient.

Sixty-five percent (9/14) of PET-activation patients had a least 1 cardiac arrest precaution measure (defibrillator pads on, backboard in place, or code-dose epinephrine drawn up) in place before intubation.

## DISCUSSION

Peri-intubation cardiac arrest is a rare but potentially fatal complication of tracheal intubation. We recognized the need for a systematic approach to hemodynamic optimization for children with critical physiologic derangements. We established a multidisciplinary improvement team and a new clinical process to decrease the risk of peri-intubation cardiac arrest. Implementation of this process has resulted in 14 successful intubations of children at high-risk for peri-intubation cardiac arrest and has not displaced PED physicians as the primary proceduralists. We feel the critical reasons for this initiative’s success are the well-established institutional RSI process that provided a foundation to build upon, and the PET checklist which may have improved situational awareness and effective communication for high-risk patient-specific mitigation strategies. This novel process may be easily extrapolated to other settings with an established RSI process but does not include physiologic risk factors for peri-intubation cardiac arrest. Similarly, for setting starting an RSI process, incorporating physiologic risk factors into the RSI checklist may be beneficial to the most high-risk patients.

Similar quality improvement studies have demonstrated a reduction in intubation related adverse events or cardiac arrest in the pediatric population. Spaeth et al^8^ demonstrated a 59% reduction in airway related cardiac arrest in pediatric patients undergoing airway management with anesthesia over 2.5 years. While this study demonstrates the potential for QI interventions to minimize patient risk, the clinical setting in which it takes place (ie, in the OR with anesthesia) is very different from emergency airway management in the PED. A PED based study conducted by Long et al,^16^ demonstrated an increase in first-pass success without hypoxemia or hypotension following the implementation of a QI bundle for emergent airway management. Our study differs in that we are specifically looking at a physiologically high-risk patient population and expanding on an already well-established RSI process.

### Limitations

This work occurred at a single large pediatric center with specialized resources that may limit generalizability. Our institutional culture, including a well-established RSI checklist and in situ simulation program, supported successful implementation but may pose challenges in other settings. Finally, the low frequency of both physiologically high-risk pediatric intubations and peri-intubation cardiac arrest limits our ability to evaluate changes in mortality and draw definitive conclusions regarding the effectiveness of our interventions. It also limits our ability to demonstrate sustainability over time. However, due to the high mortality associated with peri-intubation cardiac arrest, there is value in disseminating this ongoing work that promises to reduce mortality.

## CONCLUSIONS

We successfully developed a novel approach to mitigate the risk for peri-intubation cardiac arrest in a PED without significantly reducing key procedural opportunities for the PED team. The PET may have resulted in zero cardiac arrests during the study period, but further refinement and long-term data monitoring are needed.

## DISCLOSURE

The authors have no financial interest to declare in relation to the content of this article.
